# Field‐scale evaluation of ecosystem service benefits of bioenergy switchgrass

**DOI:** 10.1002/jeq2.70025

**Published:** 2025-04-22

**Authors:** Nictor Namoi, Cheng‐Hsien Lin, Chunhwa Jang, Daniel Wasonga, Colleen Zumpf, Muhammad Umer Arshad, Emily Heaton, DoKyoung Lee

**Affiliations:** ^1^ Department of Crop Sciences University of Illinois at Urbana‐Champaign Urbana Illinois USA; ^2^ Agroecosystem Sustainability Center, Institute for Sustainability, Energy, and Environment University of Illinois at Urbana‐Champaign Urbana Illinois USA; ^3^ DOE Center for Advanced Bioenergy and Bioproducts Innovation University of Illinois at Urbana‐Champaign Urbana Illinois USA; ^4^ Department of Soil and Environmental Sciences National Chung Hsing University Taichung Taiwan; ^5^ Environmental Science Division Argonne National Laboratory Lemont Illinois USA

## Abstract

Purpose‐grown perennial herbaceous species are nonfood crops specifically cultivated for bioenergy production and have the potential to secure bioenergy feedstock resources while enhancing ecosystem services. This study assessed soil greenhouse gas emissions (CO_2_ and N_2_O), nitrate (NO_3_‐N) leaching reduction potential, evapotranspiration (ET), and water‐use efficiency (WUE) of bioenergy switchgrass (*Panicum virgatum* L.) in comparison to corn (*Zea mays* L.). The study was conducted on field‐scale plots in Urbana, IL, during the 2020–2022 growing seasons. Switchgrass was established in 2020 and urea‐fertilized at 56 kg N ha^−1^ year^−1^. Corn management followed best management practices for the US Midwest, including no‐till and 202 kg N ha^−1^ year^−1^ fertilization, applied as urea–ammonium nitrate (32%). Our results showed lower direct N_2_O emissions in switchgrass compared to corn. Although soil CO_2_ emissions did not differ significantly during the establishment year, emissions in subsequent years were over 50% higher in switchgrass than in corn, likely due to increased belowground biomass, which was over five times higher in switchgrass. Nitrate‐N leaching decreased as the switchgrass stand matured, reaching 80% lower than in corn by the third year. Differences in ET and WUE between corn and switchgrass were not significant; however, results indicate a trend toward reduced WUE in switchgrass under drought, driven by lower aboveground biomass production. Our study demonstrates that switchgrass can be implemented at a commercial scale without negatively impacting the hydrological cycle, while potentially reducing N losses through nitrate‐N leaching and soil N_2_O emissions, and enhancing belowground C storage.

## Core Ideas

- Switchgrass and corn were compared for CO_2_ and N_2_O emissions, NO_3_‐N leaching, and evapotranspiration (ET).
- Switchgrass reduced NO_3_‐N leaching over time compared to corn.
- The ET in switchgrass was comparable to corn.
- N_2_O emissions were lower in switchgrass than corn, but CO_2_ emissions were >50% higher 2 years after establishment.
- Root‐associated respiration may drive increased CO_2_ emissions under switchgrass.

**Abbreviations**
AGB: aboveground biomass
BGB: belowground biomass
BMP: best management practices
ET: evapotranspiration
GHG: greenhouse gas
PET: potential evapotranspiration
SAF: sustainable aviation fuel
TSM: total soil moisture
UAN: urea–ammonium nitrate
VWC: volumetric water content
WUE: water‐use efficiency

## 1 INTRODUCTION

The US Department of Energy (US DOE) has recently launched the sustainable aviation fuel (SAF) Grand Challenge roadmap, aiming to produce 3 billion gallons of low‐carbon intensity SAF annually by 2030 and 35 billion gallons by 2050. This initiative envisions low carbon intensity (CI) bioenergy feedstocks, aiming for a minimum of 50% reduction in greenhouse gas (GHG) emissions across their entire life cycle (U.S. DOE et al., 2022). Purpose‐grown energy crops, including the perennial switchgrass (*Panicum virgatum* L.), are anticipated to play a significant role in achieving this target.

Designated as a model bioenergy feedstock (Wright & Turhollow, 2010), switchgrass can remain productive on marginal land while providing multiple ecosystem services, including nitrate‐N leaching reduction, erosion and runoff control, soil health improvement, C sequestration, and GHG emissions mitigation (Acharya & Blanco‐Canqui, 2018; Ferrarini et al., 2017; Martani et al., 2022). Switchgrass's ability to enhance ecosystem services is dependent on management (e.g., nitrogen [N] management), growth dynamics (primarily in its fibrous root development), and site characteristics (including climate and soil conditions) (Ferrarini et al., 2017; Jin et al., 2019; Monti et al., 2012). Soil C sequestration under switchgrass is largely attributed to the minimal soil disturbance inherent in perennial systems and the enhanced allocation of carbon to belowground structures (Agostini et al., 2015; Anderson‐Teixeira et al., 2013; Stewart et al., 2017). Nitrogen application can alter the belowground productivity and quality of biomass inputs, which affects the stabilization of organic matter (Cotrufo et al., 2013; Garten et al., 2011; Heggenstaller et al., 2009).

Nitrous oxide mitigation in switchgrass systems is achieved through avoided emissions, primarily driven by the crop's significantly lower N requirements (Ferrarini et al., 2017; Monti et al., 2012). Optimal N rates for switchgrass biomass yield rarely exceed 112 kg N ha^−1^ (Cooney et al., 2023; D. K. Lee et al., 2018; Zumpf et al., 2019), which is significantly lower than the recommended rates for Maximum Return to Nitrogen (MRTN) for corn (*Zea mays* L.) following corn (215–225 kg N ha^−1^) and for corn following soybean (*Glycine max* L.) (190–220 kg N ha^−1^) (Nafziger, 2020). In addition to reduced N inputs compared to annuals, the extensive root systems of switchgrass help minimize N losses by enhancing nutrient recovery, reducing leaching, and limiting N availability for microbial‐mediated N_2_O production (Abalos et al., 2018; Daly et al., 2022; Hussain et al., 2019; Monti et al., 2012).

Soil characteristics and weather conditions significantly influence soil GHG emissions. Typically, N_2_O “hot moments” occur after fertilization and precipitation, when available N and anaerobic conditions align (Anthony & Silver, 2023; Daly et al., 2022; Zhang et al., 2021). Favorable weather conditions can decrease residual soil N due to increased plant uptake. Although denitrification is the primary source of N_2_O emissions, nitrification also plays a significant role under dry and aerobic conditions (Van Groenigen et al., 2010). Soil CO_2_ emissions are influenced by temperature and fertilization. Vegetation can further elevate emissions through root‐associated respiration (Kuzyakov, 2006; Oertel et al., 2016; Yue et al., 2023). Additionally, soil texture, organic matter content and quality, and the presence of Fe/Al oxides affect heterotrophic respiration, contributing to soil CO₂ efflux (Chen et al., 2019; Kuzyakov, 2010; Ren et al., 2022).

Previous studies have separately investigated GHG emissions, nitrate‐N leaching, and water use (hereafter referred to as evapotranspiration [ET]) in switchgrass in the US Midwest (Anderson‐Teixeira et al., 2013; Hamilton et al., 2015; Hussain et al., 2019; McIsaac et al., 2010). However, the site‐specific nature of these outcomes underscores the need for further studies. Furthermore, previous studies focused on older switchgrass cultivars developed for forage production. New bioenergy varieties have been developed through lowland × highland ecotype crosses or by improving lowland ecotypes. The advanced bioenergy cultivars have better establishment, faster growth, later flowering, enhanced winter survivability, and improved biomass yield (Casler, 2012; Casler et al., 2018; M. S. Lee et al., 2024; Vogel et al., 2014). More studies are therefore necessary to evaluate the ecosystem service outcomes associated with bioenergy switchgrass (defined as varieties bred for use as bioenergy feedstocks). In this study, we evaluated GHG emissions, ET, water‐use efficiency (WUE), and NO_3_‐N leaching for the recent switchgrass cultivar Independence (M. S. Lee et al., 2024) compared to corn, both managed under best management practices (BMP) for Illinois. We hypothesized that switchgrass would have lower direct N_2_O emissions than corn due to reduced N application rates and more efficient N recovery, but higher soil CO_2_ emissions from root‐associated respiration (Anderson‐Teixeira et al., 2013; Monti et al., 2012). Additionally, we anticipated that Independence switchgrass would exhibit higher WUE, driven by increased biomass yield and reduced ET. Finally, we expected lower NO_3_‐N leaching under switchgrass compared to corn due to improved N recovery facilitated by increased belowground biomass (BGB).

## 2 MATERIALS AND METHODS

### 2.1 Site and field management

The field experiment was conducted under rainfed conditions at the University of Illinois Energy Farm in Urbana, Champaign County, Illinois (40.068641° N, −88.190864° W), on land that is categorized as marginal—that is, inherently low crop productivity index, frequent ponding, flooding, and poor drainage (Cacho et al., 2023). The field had a long history of various perennial grasses, followed by a soybean‐corn rotation in 2018 and 2019, respectively. The site has a mean historical (1989–2021) annual precipitation of 940 mm and a mean annual temperature of 11°C. The soil consisted of 83% Flanagan silt loam (mesic Aquic Argiudolls) and 17% Drummer silt clay loam (mesic Typic Endoaquolls) (Soil Survey Staff, 2023). The baseline soil properties are shown in Table 1.

**TABLE 1:** Pretrial soil characteristics at the University of Illinois Energy Farm in Urbana, IL (0–90 cm).

Depth (cm)	SOM (%)	Total C (%)	pH	P	K	Ca	Mg	S	CEC (cmolc kg^−1^)	BD (g cm^−3^)
				Extractable^a^ (mg kg^−1^)						
0–10	3.31	1.64	6.11	25.5	150	1720	287	5.1	13.3	1.0
10–20	3.09	1.57	6.13	16.4	98	1958	235	5.5	14.1	1.2
20–30	2.89	1.35	6.34	11.6	83	2102	287	4.8	14.7	1.1
30–60	2.56	1.04	6.63	7.6	85	2185	411	3.9	15.6	1.1
60‐90	1.87	0.6	6.98	4.9	86	1967	473	3.3	14.5	1.2

Abbreviations: BD, bulk density; CEC, cation exchange capacity; SOM, soil organic matter.

^a^ Extracted using the Mehlich 3 soil test.

The experimental plots (∼0.2 ha each) were organized using a Randomized Complete Block Design (RCBD) with three replicates (*n* = 6) comparing soil GHG (CO_2_ and direct N_2_O) emissions, ET, WUE, and NO_3_‐N leaching between switchgrass and corn. Both crops were managed according to BMP for central Illinois. A summary of the major field activities and their timing is shown in Table S1 and given in greater detail in Cacho et al., 2023, and Hamada et al., 2021. Independence variety switchgrass was established under no‐till. Planting was accomplished using a John Deere 750 no‐till drill in the spring of 2020 at a seeding rate of 323 pure live seed (PLS) m^−2^ rate and a 19‐cm row spacing. Preemergence weed control involved a tank mix of 3.0 kg a.i. ha^−1^ Quinclorac (3,7‐dichloro‐8‐quinolinecarboxylic acid) and 2.2 kg a.i. ha^−1^ Atrazine (2‐chloro‐4‐ethylamine‐6‐isopropplyamino‐s‐triazine), followed by postemergence application of 1.68 kg a.i. ha^−1^ 2,4‐D. The switchgrass plots were unfertilized in the establishment year, consistent with BMP guidelines (D. Lee et al., 2014; Mitchell et al., 2014), and later received 56 kg N ha^−1^ of broadcasted urea (46‐0‐0) annually each spring. For the continuous corn plots, glyphosate‐tolerant corn (Dekalb DKC 60–87 RIB) was planted in mid‐May at a seeding rate of 84,000 seeds ha^−1^ with standard row spacing of 0.76 m (Winans et al., 2021), using a John Deere 1700 Max Emerge XP corn planter. All field management practices followed the BMP guidelines for Illinois, including no‐till, N application by direct injection at 202 kg N ha^−1^ as urea–ammonium nitrate (UAN) (32%), preemergence Dual Magnum Herbicide (S‐metolachlor) at 2.5–3.2 kg a.i. ha^−1^, and postemergence Roundup (glyphosate) at 0.84 kg a.i. ha^−1^.

**Core Ideas**

- Switchgrass and corn were compared for CO_2_ and N_2_O emissions, NO_3_‐N leaching, and evapotranspiration (ET).
- Switchgrass reduced NO_3_‐N leaching over time compared to corn.
- The ET in switchgrass was comparable to corn.
- N_2_O emissions were lower in switchgrass than corn, but CO_2_ emissions were >50% higher 2 years after establishment.
- Root‐associated respiration may drive increased CO_2_ emissions under switchgrass.

### 2.2 Plant and soil sampling

Corn and switchgrass were harvested after maturity and after a killing frost, respectively. For corn, the entire corn plot was harvested using a John Deere 9610 Combine, and the fresh grain yield was adjusted for the machine‐recorded moisture percentage (averaging ∼18%) to obtain the dry corn grain yield. The aboveground biomass (AGB) yield was then estimated using the standard corn harvest index of 0.545 (Vogel & Below, 2019). Switchgrass was hand‐harvested using a sickle knife at ≈15 cm cutting height from three random 1 m × 1 m quadrats, which were randomly placed within each plot at the start of the growing season, prior to emergence. Bundle fresh weight was recorded. Dry biomass yield was calculated by adjusting for the moisture percentage obtained from oven‐drying a subsample for 72 h at 60°C and scaling the results to kg ha^−1^.

Soil C and BGB were measured in December 2023. Duplicate 90‐cm soil cores were collected using a Giddings probe from three locations in each plot—one from within the crop row at the plant crown and the other from the interrow space between corn and switchgrass rows. Both samples were sectioned into five intervals (0–10, 10–20, 20–30, 30–60, and 60–90 cm). Approximately 100 g of root‐free soil was extracted from each interrow core, composited, and used to measure total soil C. Total C was determined using a LECO FP‐2000 CN analyzer (LECO Corporation). Roots from the crop‐row and between‐row cores were separated by washing the soil through a 0.5‐mm sieve with water, manually picking them out, oven‐drying for 48 h at 60°C, and weighing to determine dry root biomass. Total belowground biomass (BGB) was calculated as the average of the crop‐row and between‐row samples.

### 2.3 Soil CO_2_ and direct N_2_O emissions measurements

Soil GHG fluxes were measured over three growing seasons (2020–2023). Sampling began at switchgrass emergence, with a delayed start in 2020, as full switchgrass emergence after seeding was not achieved until mid‐June, after which sampling occurred weekly until July and then focused on the 1–2 days after fertilization and 2–4 days following significant rainfall events. After July, sampling was conducted weekly throughout the remainder of the growing season in 2020, 2022, and 2023. In 2021, the post‐July sampling was conducted at least once every 3 weeks during the growing season. Gas samples were collected from static chambers installed at six locations within each plot (*N* = 36) during 2020–2021. In 2022, we reduced the number of locations to three per plot (*N* = 18). Chamber construction followed guidelines established by the USDA Agricultural Research Service GRACEnet project protocol (Parkin & Venterea, 2010). The static chamber consisted of a ground anchor and a top chamber. Circular PVC columns with an internal diameter of 20.3 cm and a height of 10 cm were utilized as ground anchors, and these were installed to achieve 6.5 cm of aboveground height. White, dome‐shaped PVC end caps with reflective properties were used for the top chamber. A combination of insulation foam and Santoprene material was used in the chamber interior, and stainless‐steel hose clamps were employed to fasten it, ensuring an airtight seal with the anchor. In total, a headspace volume of 0.005 m^3^ was achieved. The soil CO_2_ and direct N_2_O fluxes were measured instantaneously using a closed‐loop arrangement with the DX4040 and GT5000 Terra portable FTIR gas analyzers (Gasmet Technologies) (Martinez & Ardón, 2021; Pakorn et al., 2012). The gas concentrations were sampled at 20‐s intervals for 5 min following the placement of the chamber. The gas fluxes were computed using the ideal gas law and linear regression (Collier et al., 2014; Parkin & Venterea, 2010) and then scaled to estimate hourly and daily emissions. Daily emissions between consecutive sampling events were calculated by linear interpolation, and the cumulative growing season was computed.

### 2.4 NO_3_‐N leaching measurements

Nitrate‐N leaching was monitored using MacroRhizon suction pore water samplers (Rhizosphere) (Dewey et al., 2023; Zumpf et al., 2017) that were installed adjacent to the gas chambers at 30‐ and 90‐cm depths. After substantial rainfall, leachate samples were collected using evacuated syringes throughout the growing season. The NO_3_‐N concentration was determined through spectrophotometry using an automated wet chemistry analyzer (Smart Chem 200 Discrete Auto Analyser; Systea).

### 2.5 Water use and water‐use efficiency estimation

The ET was estimated using the water balance approach based on the daily decrease in volumetric water content (VWC) (Hamilton et al., 2015; McIsaac et al., 2010; Shah et al., 2012). Briefly, VWC changes in each plot were monitored using Teros 12 moisture sensors (Meter Group) installed at 30, 60, and 90 cm and connected to a ZL6 Data Logger (Meter Group). Two sensors, one from a corn plot and one from a switchgrass plot, failed in 2020 and were excluded from the analysis. Total soil moisture (TSM) over the entire 90‐cm profile was calculated using the trapezoidal formula for nonuniform increments (Zumpf et al., 2017). Subsurface drainage losses are assumed to be the TSM changes between midnight (0000 h) and 0400 h, as no ET is expected between this time interval. Hourly and subsequently daily subsurface drainage losses (*Q*) were calculated, and finally, daily ET (mm) was calculated as the change in TSM at midnight between two consecutive days (TSM*_j_* and TSM*_j_*_+1_) adjusted for drainage (Shah et al., 2012) (Equation 1).

(1) $$\mathrm{ET}=\mathrm{TS}M_{j}-\mathrm{TS}M_{j+1}+Q$$

This procedure assumes that daily net VWC depletion is due to ET. Gains in VWC are effectively due to precipitation events. Thus, any TSM values above field capacity (FC) were screened out as we assumed that all precipitation above FC was lost through drainage (subsurface drainage or surface runoff) (McIsaac et al., 2010). The FC was estimated as the average TSM content in the 30 days preceding switchgrass emergence because the soil profile fully recharges during winter (Hamilton et al., 2015). Our average profile VWC of 0.38 cm^3^ cm^−3^ at FC is comparable to McIsaac's values (0.34 cm^3^ cm^−3^) (McIsaac et al., 2010) and the county average of 0.36 cm^3^ cm^−3^ (WARM‐CN, 2023), translating to a TSM of 33.90 cm. We excluded days with a net increase in TSM, days with precipitation events and the subsequent 3 days, as well as days when moisture exceeded FC. The remaining data were assumed to represent actual ET, reflecting soil moisture depletion.

Potential evapotranspiration (PET) was calculated using the FAO 56 Penman–Monteith equation utilizing the minimum data requirements described in Allen et al. (1998). The maximum and minimum temperature, maximum and minimum relative humidity, wind speed, atmospheric pressure, and sunshine hours were obtained from the Champaign‐Urbana almanacs (Illinois State Climatologist, 2023). The ET to PET ratio was established, and on days with excluded data, ET was approximated by applying the latest ET:PET ratios to PET values (McIsaac et al., 2010). The WUE was calculated by dividing AGB by the cumulative ET over the growing season.

### 2.6 Statistical analysis

The data were analyzed using the generalized linear mixed model approach in R version 4.3.3 (R Core Team, 2024). The R package “lme4” was used (Bates et al., 2015). Fixed factors were crop type (crop), growing season (year), and sampling depth (depth), while random effects were the blocks. The *p*‐values for fixed effects were obtained using the “lmerTest” package (Kuznetsova et al., 2017), and where significant (*p* = 0.05), the “emmeans” package was used for pairwise comparison using the Tukey adjustment method (*p* = 0.05) (Lenth, 2021).

## 3 RESULTS AND DISCUSSION

### 3.1 Weather and growing conditions

The weather patterns observed in 2020 and 2021 aligned with long‐standing trends (Table 2). In 2022, at Urbana, despite temperatures remaining near long‐term averages, precipitation was low between April and July, leading to 25% and 30% lower growing season and total precipitation than long‐term averages, respectively.

**TABLE 2:** Monthly precipitation and average monthly temperature conditions, and their 30‐year averages in Urbana, IL.

Month	Temperature (°C)				Precipitation (mm)			
	2020	2021	2022	30‐Year average	2020	2021	2022	30‐Year average
Jan.	−0.2	−1.1	−5	−4	82.8	45	11.2	52.1
Feb.	−0.9	−5.6	−2.1	−1.7	18.5	35.1	30.2	51.3
Mar.	6.3	7.5	5.7	4.4	70.9	109	113.5	69.9
Apr.	9.5	11.6	10.4	11.1	70.1	48.8	69.1	96.3
May	15.5	15.5	19.2	16.9	106.4	89.7	95.5	115.6
June	23.2	23.5	24	22.3	129.8	166.6	32	105.9
July	24.9	22.9	24.1	23.9	95.5	105.9	63	110.2
Aug.	22.3	23.5	22.3	23	39.6	50.5	88.4	91.7
Sept.	18	20.8	18.8	19	68.3	78.2	57.4	83.3
Oct.	11.2	15.5	12	12.2	49	136.9	57.9	79.8
Nov.	7	4.4	5.6	5.2	59.4	30.5	45.7	88.9
Dec.	0.6	4.7	−0.2	−1.7	33.3	55.9	65.5	64
Average	11.5	11.9	11.2	10.9	823.6	952.1	729.4	1009
Growing season	18.9	19.6	19.8	19.4	509.7	539.7	405.4	603

*Note*: The data were obtained from Champaign‐Urbana Willard Airport Station.

### 3.2 Above‐ and belowground crop biomass yield and soil C

Crop × year interactions (*p* = 0.003) affected AGB yield (Table 3). Switchgrass AGB was about one‐third of corn's yield in the establishment year (2020) but increased in subsequent years as roots developed, which is consistent with established trends (Mclaughlin & Kszos, 2005; Parrish & Fike, 2005). Differences in AGB were not significant in 2021 and 2022. Similar yield ranges have been reported for both crops (Dohleman & Long, 2009; Dohleman et al., 2012; Kantola et al., 2022; Masters et al., 2016; Varvel et al., 2008). While not statistically significant, we observed a 25% decline in switchgrass AGB in 2022 compared to 2021, likely due to early‐season drought reducing stomatal conductance and CO_2_ assimilation (Hartman et al., 2012; Tejera‐Nieves et al., 2023).

**TABLE 3:** Total aboveground biomass yield (grain + stover), estimated water use (ET), and water‐use efficiency (WUE) for switchgrass and corn during the 2020–2022 growing seasons at Urbana, IL.

	Aboveground (kg ha^−1^)		Estimated ET (mm)		WUE (kg ha^−1^ mm^−1^)	
	Corn	Switchgrass	Corn	Switchgrass	Corn	Switchgrass
**Year**						
2020	11,682a	3794b	161a	165a	87a	31a
2021	10,992ab	16,722a	193a	327a	58a	79a
2022	10,056ab	12,400ab	254a	287a	47a	50a
	10,910A	10,972A	203A	260A	64A	53A
**Source of variation**						
Crop		0.963		0.274		0.6402
Year		0.007		0.181		0.7999
Crop × year		0.003		0.501		0.1867

*Note*: Lowercase letters show crop × year interactions, while uppercase letters indicate the main effects of crop.

The BGB yield was influenced by the crop × depth (*p* = 0.002) interactions (Table 4). Switchgrass accumulated 75% of its BGB in the upper 0–10 cm. Switchgrass BGB was about six times that of corn at the 0‐ to 10‐cm depth (*p* < 0.001). Though not different across the other depths, the total BGB in switchgrass was five times that of corn. These findings align with previous research showing increased C allocation belowground in perennials compared to annuals, up to 80% of which is in the top 30 cm (Anderson‐Teixeira et al., 2013; Heggenstaller et al., 2009; Ma et al., 2000). Root‐to‐shoot ratios of up to 3 have been reported in switchgrass (Kibet et al., 2016; Sainju et al., 2017).

**TABLE 4:** Root biomass accumulation (kg ha^−1^) and soil total carbon (g kg^−1^) in corn and switchgrass during 2023 after 3 and a half years of management.

	Root biomass (kg ha^−1^)		Soil total C (g kg^−1^)	
Depths	Corn	Switchgrass	Corn	Switchgrass
0–10	3520b	17,840a	18.44ab	21.56a
10–20	200b	1340b	15.48bc	14.88bc
20–30	120b	680b	14.82bc	12.73cd
30–60	90b	2570b	9.46de	7.2ef
60–90	110b	1090b	4.79f	4.63f
0–90^a^	4040B	23,530A	12.60A	12.20A
Crop	0.002		0.23	
Depth	<0.001		<0.001	
Crop × depth	0.002		0.009	

*Note*: Lowercase letters show crop × depth interactions, while uppercase letters indicate the main effects of crop.

^a^ Total root biomass and average SOC over 0‐ to 90‐cm depth.

While crop × depth interactions were significant (*p* = 0.009), differences in soil C were not statistically different between corn and switchgrass at each depth, aside from the expected decrease with depth (Table 4). This is contrary to our expectations of greater C accumulation in switchgrass. These results are, however, not unprecedented, as no‐till corn with at least 70% residue retention has been shown to improve soil C (Qin et al., 2016; Stewart et al., 2015). Furthermore, while not statistically different, soil C was numerically higher in switchgrass at the surface 0‐ to 10‐cm depth, which can be attributed to the slow accrual of soil C and high spatial and temporal variability, likely hindering the detection of significant differences between treatments (Guan et al., 2023; Stewart et al., 2007; Wuest & Durfee, 2024). Thus, the increase in C stocks at the 30‐ to 60‐cm depth likely resulted from more soil mass rather than C concentration (Table S2).

### 3.3 Soil CO_2_ and N_2_O direct N_2_O emissions

**FIGURE 1:**
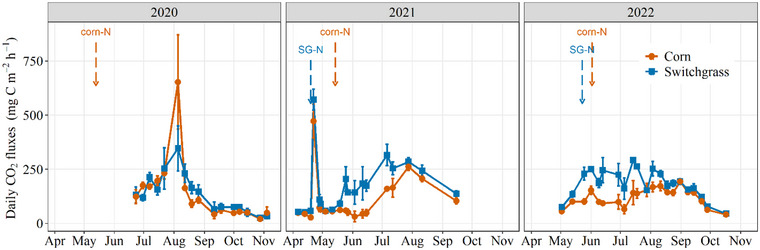
Daily CO_2_ fluxes in Urbana, IL, during the 2020 (switchgrass establishment year) to 2022 growing seasons Arrows indicate the timing of N application to corn (red) and switchgrass (blue). Errors bars represent standard errors.

**FIGURE 2:**
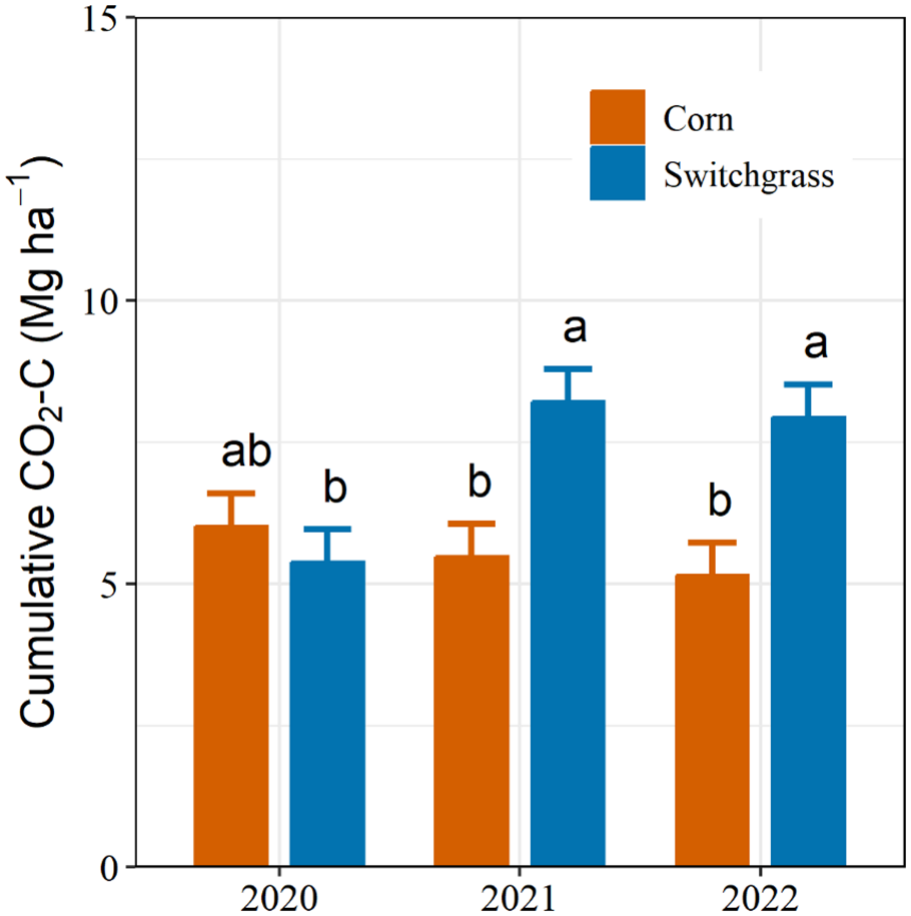
Cumulative CO_2_‐C emissions from corn and switchgrass in Urbana, IL, during the 2020 (switchgrass establishment year) to 2022 growing seasons. Lowercase letters indicate differences from crop × year interactions. Error bars indicate Standard errors of the mean.

In‐season soil CO_2_ fluxes were comparable in corn and switchgrass, though corn had higher fluxes during peak periods (Figure 1). In 2021 and 2022, switchgrass showed more stable and slightly higher fluxes, with emissions generally following temperature trends: low in spring, rising in summer, and declining in fall. The results show crop × year interactions affected cumulative growing season soil CO_2_ emissions (*p* = 0.006). While the cumulative soil CO_2_ emissions did not differ between switchgrass and corn in the establishment year (Figure 2), the emissions during the 2021 and 2022 growing seasons were higher (>50%) in switchgrass than in corn. We attribute the increase to root‐associated respiration from the greater switchgrass BGB (i.e., root autotrophic respiration and/or heterotrophic respiration through rhizosphere priming and microbial decomposition of root exudates), which may account for up to 55% of the total respiration from switchgrasses (Anderson‐Teixeira et al., 2013; Kuzyakov, 2006; von Haden, 2017).

Increased heterotrophic respiration from root‐free soil cannot be ruled out and may be due to enhanced carbon mineralization, particularly in the surface layer (0–10 cm), where switchgrass showed an increase in soil carbon. Previous studies have reported increased microbial biomass turnover and mineralization rates in switchgrass systems (Agostini et al., 2015; D. K. Lee et al., 2007; Stewart et al., 2017; von Haden et al., 2019a). Since BGB can vary between switchgrass cultivars (Ma et al., 2000; Stewart et al., 2017), further research is needed to determine how these BGB differences might influence the relative contributions of heterotrophic and autotrophic respiration to overall soil CO_2_ emissions.

**FIGURE 3:**
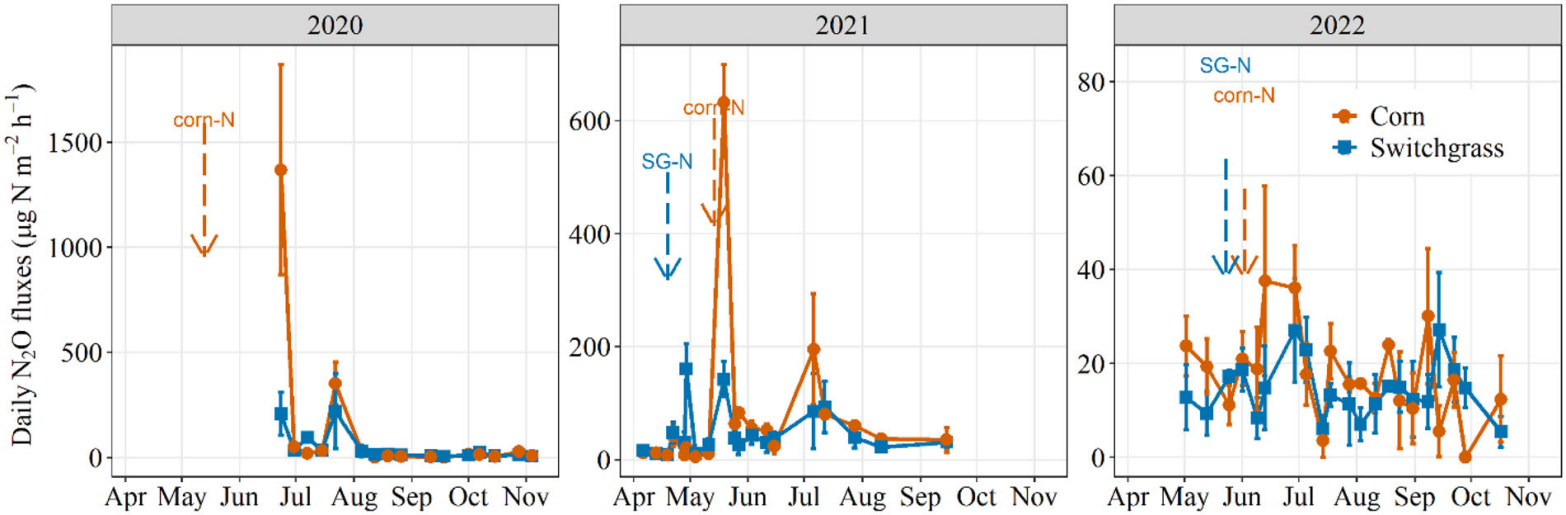
Daily N_2_O fluxes in Urbana, IL, during the 2020 (switchgrass establishment year) to 2022 growing seasons. Arrows indicate the timing of N application to corn (red) and switchgrass (blue). Errors bars represent standard errors.

**FIGURE 4:**
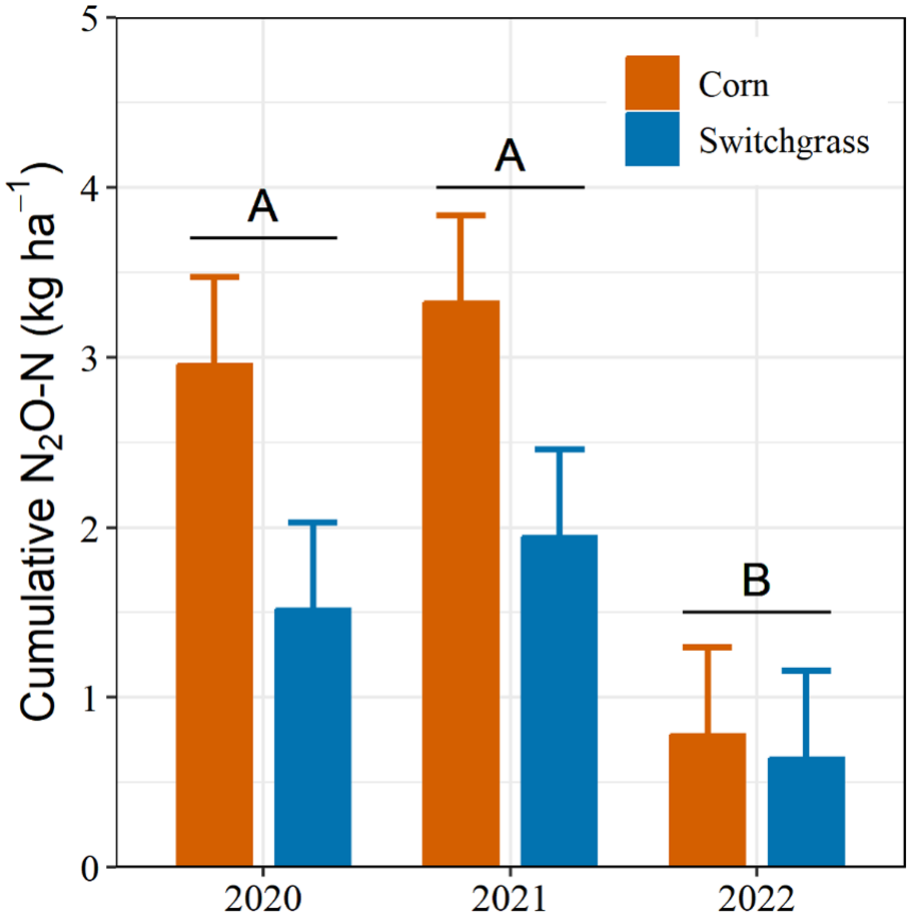
Cumulative N_2_O‐N losses from corn and switchgrass in Urbana, IL, during the 2020 (switchgrass establishment year) to 2022 growing seasons. Uppercase letters indicate main effects of year. The main effects of crop were significant (*p* = 0.05), while crop × year interactions were not significant. Error bars indicate standard errors of the mean.

Temporal direct N_2_O emissions trends generally followed expectations, peaking after fertilizer application and rainfall events (Figure 3), highlighting the influence of denitrification on direct soil N_2_O emissions. Fertilizer provides mineral N, and increased soil moisture promotes anaerobic conditions that enhance denitrification (Butterbach‐Bahl et al., 2013; Chantigny et al., 2016). The peaks in direct N_2_O fluxes were less pronounced in 2022 than in the previous seasons, however, and cumulative emissions were >65% lower (*p* = 0.02; analysis of variance [ANOVA] table in Table S3) than in 2020 and 2021 (Figure 4). We attribute these results to persistent drought conditions from April to July, which limited denitrification by reducing mineral N availability and microbial activity across all N_2_O production pathways (Gundersen et al., 2012; Harris et al., 2021; Oertel et al., 2016). The denitrification pathway has been found to account for up to 70% of N_2_O emissions in soils with as low as 20% water‐filled pore space (Harris et al., 2021). The dominance of denitrification is attributed to strong moisture retention in anoxic microsites in the soil.

We acknowledge that unmonitored nitrogen losses via ammonia (NH₃) volatilization may have contributed to the absence of a detectable N_2_O emission peak in the switchgrass plots during 2022. Dry, windy conditions, high soil pH, and the presence of urease from crop residue can exacerbate volatilization losses, especially when urea‐based fertilizers are broadcast (Del Moro et al., 2017; Hargrove, 1988).

Nevertheless, switchgrass had nearly half (*p* = 0.014) the soil N_2_O emissions of corn (Figure 4), likely due to higher N application rates in corn. Additionally, the extensive fibrous rooting system of switchgrass likely results in greater uptake of the available N, reducing the potential for transformation to N_2_O (Abalos et al., 2018; Daly et al., 2022; Van Groenigen et al., 2010).

### 3.4 NO_3_‐N leaching

**FIGURE 5:**
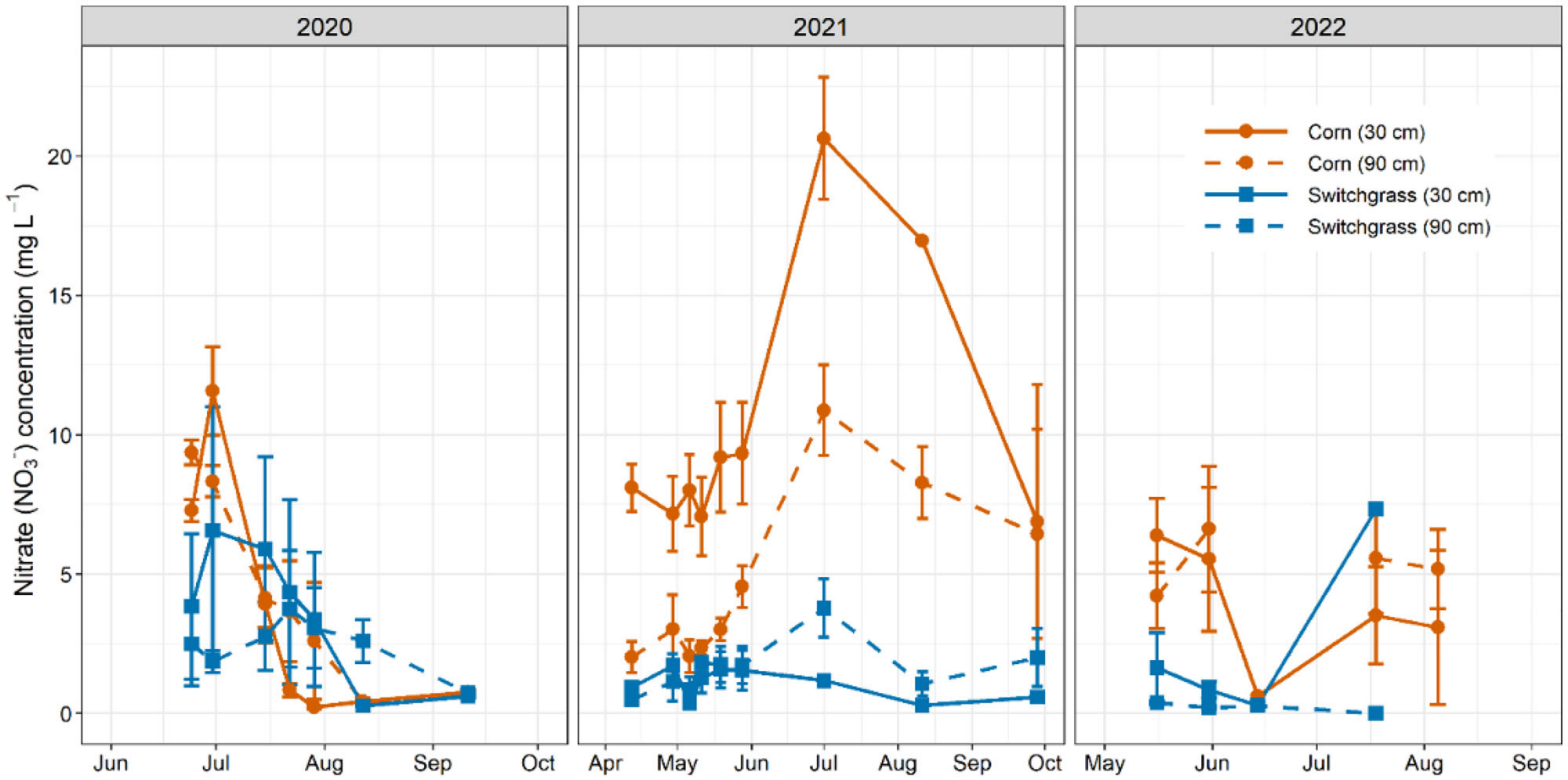
Concentrations of NO_3_‐N in corn and switchgrass in soil leachates at 30 cm (solid lines) and 90 cm (dashed lines) during the 2020 (establishment year) to 2022 growing season at Urbana, IL. Errors bars represent standard errors.

Year × depth × crop (*p* = 0.027) interactions affected NO_3_‐N concentration in leachate (Table S4). In 2020, NO_3_‐N concentrations did not differ (at *p* = 0.05) between corn and the unfertilized switchgrass at 30‐ and 90‐cm depths (Figure 5). These results were unexpected and may indicate the presence of residual NO_3_‐N from previous corn or, more likely, inherently high soil mineralization rates. Previous studies have also attributed NO_3_‐N leaching from unfertilized soils to high organic matter and N mineralization rates in the midwestern soils (Jungers et al., 2019; Leon et al., 2023). We suggest that switchgrass, still developing roots during establishment, was less able to recover NO_3_‐N, while corn utilized it more effectively, especially at 30 cm. This may explain concentrations in corn being twice as high but not statistically different from switchgrass at 90 cm.

While not statistically significant, switchgrass plots exhibited a consistent decline in NO_3_‐N concentrations over time, particularly at the 90‐cm depth. By 2022, average NO_3_‐N levels were 11‐fold lower compared to 2020, suggesting minimal groundwater leaching. These results align with our expectation that the increasing root density (Table 4) would enhance NO_3_‐N recovery, thereby lowering NO_3_‐N under switchgrass. In contrast, NO_3_‐N in corn at 90 cm remained similar throughout the 3 years. Overall, when averaged across depths, switchgrass plots had approximately 20% lower NO_3_‐N concentrations than corn in 2020, but this difference increased to more than 80% in the subsequent years. The potential for NO_3_‐N loss reduction in switchgrass has been shown to increase over time, typically as the stand matures, potentially reaching up to 80% within 3 years due to increased root uptake (Hussain et al., 2019; Massey et al., 2020; Smith et al., 2013).

### 3.5 Water use and water‐use efficiency

**FIGURE 6:**
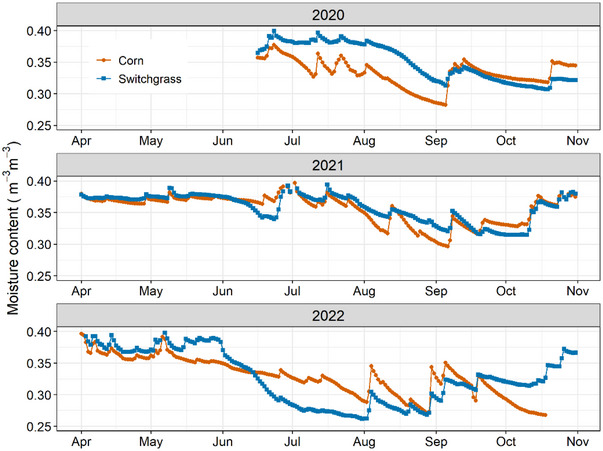
Mean volumetric soil moisture content (0–90 cm) during the 2020 (establishment year) to 2022 growing seasons. Soil moisture measurements during the establishment year began in June in both corn and switchgrass plots.

In 2020, TSM was lower in corn than in switchgrass until September, when the gap narrowed, and ultimately there was higher soil moisture in corn (Figure 6). This narrowing of differences and the increase in TSM in corn can be attributed to reduced transpiration as corn reached maturity, while switchgrass continued to actively transpire until the onset of senescence later in the season (Hendrickson et al., 2013; Zeri et al., 2013; Zumpf et al., 2017). The elevated TSM observed across all plots in early spring (2021–2022) can be attributed to soil water recharge, driven by reduced ET and increased snowmelt recharge (Abraha et al., 2020; von Haden et al., 2019b). The TSM in 2021 was comparable between corn and switchgrass until August, likely because of the frequent precipitation balancing ET. In 2022, however, we observed a drought‐induced decline in TSM across all plots between June and August, when rainfall was more than 25% below the long‐term growing season averages (Table 1). The decline was steeper in switchgrass, with TSM averaging nearly 10% lower than in corn (Figure 6). The steeper decline in TSM in switchgrass may be due to its rapid root expansion, with deeper and more divergent fine roots that enhance moisture depletion. Drought‐induced surface water limitations exacerbate TSM disparities, as switchgrass relies more heavily on subsurface moisture (Abraha et al., 2020; Joo et al., 2017; von Haden et al., 2019b). Increased TSM observed under switchgrass (averaging 32.35 ± 3.0 mm) compared to corn (averaging 30.08 ± 3.0 mm) during the 2020 growing season is likely due to its less extensive root growth and limited AGB production during the establishment phase (Table 3), resulting in slower soil moisture depletion (Abraha et al., 2020; Mclaughlin & Kszos, 2005; Parrish & Fike, 2005).

Our ET data (Table 3) are over twofold lower than that observed in similar water balance studies in the US Midwest (Hamilton et al., 2015; Stephenson et al., 2021), likely due to site‐specific differences. Studies from central Illinois and Wisconsin, showing ET of 262–352 mm for corn and 283–355 mm for switchgrass, are, however, comparable to our data (McIsaac et al., 2010; Parish et al., 2019). Even more surprising, our data were about three times lower than satellite‐based measurements at the same location (Zumpf et al., 2023). We partially attribute this disparity to methodological differences. However, given the substantial variance observed, future studies may need to prioritize achieving better synchrony between the two approaches. Overall trends were similar, however, with switchgrass ET trending higher but not statistically different than corn (Table 3) and lower ET in drought years for switchgrass. The studies noted that ET differs between annuals and perennials when the growing season is substantially longer or when switchgrass is grown on land with a high water table and subsurface drainage (Hamilton et al., 2015; Stephenson et al., 2021; Zumpf et al., 2017). The ET of switchgrass is expected to increase over time as the grass becomes more established (Abraha et al., 2020; Zumpf et al., 2023). Studies on high‐yielding, more mature stands (>3 years) are therefore necessary to evaluate the long‐term impact of switchgrass on hydrology.

Similar to ET, WUE did not significantly differ between corn and switchgrass. Nevertheless, overall trends showed biomass yield as the primary driver of WUE (Hamilton et al., 2015; Zeri et al., 2013), with reduced WUE in switchgrass during establishment and a significant increase the next year. Despite increased maturity, the decline in WUE in 2022 compared to 2021 (Table 3) can also be attributed to reduced biomass yields caused by drought conditions during the growing season (Hartman et al., 2012; Tejera et al., 2023). Future studies should investigate whether older stands can maintain high WUE under drought conditions.

## 4 CONCLUSIONS

Demonstrating the contribution of perennial bioenergy crops to ecosystem services provision is crucial for promoting their adoption and ensuring sustainable production. The study results showed that switchgrass emits about half the direct soil N_2_O emissions of corn, but root respiration might contribute to increased soil CO_2_ emissions. The data also suggest that NO_3_‐N leaching declined over time in switchgrass.

Neither ET nor WUE showed any significant difference between corn and switchgrass, although the general trend was toward higher WUE in switchgrass as biomass increased over time. More time is needed to confirm whether WUE will be enhanced with increasing stand maturity, as the drought conditions undoubtedly impacted the results. While rooting behavior might play a role, we suggest that the lower N fertilizer requirement of switchgrass is likely the primary factor contributing to its reduced N_2_O emissions compared to corn. Overall, our data suggest the potential for BGB carbon to demonstrate improved C storage in perennial crop landscapes. Future studies should also assess whether root biomass differs between various switchgrass cultivars and their impact on soil CO_2_ emissions. We also recommend further research to characterize rhizosphere respiration in perennial systems, specifically partitioning rhizosphere priming and autotrophic root respiration. Overall, our study highlights the role of purpose‐grown herbaceous species in meeting energy objectives, concurrently mitigating GHG emissions, and minimizing NO_3_‐N leaching without detrimental effects on hydrological processes.

## AUTHOR CONTRIBUTIONS

**Nictor Namoi**: Conceptualization; data curation; formal analysis; investigation; methodology; writing—original draft; writing—review and editing. **Cheng‐Hsien Lin**: Conceptualization; data curation; investigation; methodology; project administration. **Chunhwa Jang**: Formal analysis; writing—original draft; writing—review and editing. **Daniel Wasonga**: Investigation; project administration. **Colleen Zumpf**: Investigation; writing—review and editing. **Muhammad Umer Arshad**: Formal analysis. **Emily Heaton**: Supervision; writing—review and editing. **DoKyoung Lee**: Conceptualization; investigation; project administration; resources; writing—review and editing.

## CONFLICT OF INTEREST STATEMENT

The authors declare no conflicts of interest.

## Supporting information




**Supplemental Table S1**. Main field activities and their timings during the 2020‐2022 growing seasons in Urbana, IL.
**Supplemental Table S2**. Soil total carbon stock (Mg C ha^−1^) in Corn and switchgrass during 2023 after three and a half years of management.
**Supplemental Table S3**: Analysis of variance (ANOVA) showing effects of main factors of the Crop and growing season (Year) on cumulative greenhouse gas emissions (CO_2_‐C and N_2_O‐N) at Urbana, IL during 2020‐22022.
**Supplemental Table S4**: Nitrate‐N concentrations (means ± SE) in switchgrass and corn plots at 0‐30 cm and 30‐ 90 cm depths in Urbana, IL, during the 2020‐2022 growing seasons.
