# Supplementary material for: Field‐scale evaluation of ecosystem service benefits of bioenergy switchgrass
Source: J Environ Qual. 2025 Apr 22;54(3):576–89. doi: 10.1002/jeq2.70025 (PMC12065062; doi:10.1002/jeq2.70025)
Supplement: Supplementary file 1 — Supplemental Table S1. Main field activities and their timings during the 2020‐2022 growing seasons in Urbana, IL. Supplemental Table S2. Soil total carbon stock (Mg C ha−1) in Corn and switchgrass during 2023 after three and a half years of management. Supplemental Table S3: Analysis of variance (ANOVA) showing effects of main factors of the Crop and growing season (Year) on cumulative greenhouse gas emissions (CO2‐C and N2O‐N) at Urbana, IL during 2020‐22022. Supplemental Table S4: Nitrate‐N concentrations (means ± SE) in switchgrass and corn plots at 0‐30 cm and 30‐ 90 cm depths in Urbana, IL, during the 2020‐2022 growing seasons. [file JEQ2-54-576-s001.docx]

Field-Scale Evaluation of Ecosystem Service Benefits of Bioenergy-Type Switchgrass

Nictor Namoi ^1,2,3^, Cheng-Hsien Lin^1,4^, Chunhwa Jang ^1,2,3^, Daniel Wasonga^1,2^, Colleen Zumpf ^5^, Muhammad Umer Arshad^1,2^, Emily Heaton^1,2,3^, DoKyoung Lee ^1,2,3,5*^

^*^Corresponding author: DoKyoung Lee, leedk@illinois.edu

Number of supplemental tables: 4

Supplemental Table S1. Main field activities and their timings during the 2020-2022 growing seasons in Urbana, IL

| Year | Activity | Urbana | | |
| --- | --- | --- | --- | --- |
|  |  | Switchgrass |  | Corn |
| 2020 | Seeding | May 30 |  | May 13 |
|  | N application | -- |  | May 13 |
|  | Harvest | Dec 4 |  | Oct 14 |
| 2021 | Seeding | -- |  | May 14 |
|  | N application | Apr 19 |  | May 15 |
|  | Harvest | Dec 2 |  | Oct 4 |
| 2022 | Seeding | -- |  | May 20 |
|  | N application | May 24 |  | June 2 |
|  | Harvest | Oct 19 |  | Oct 17 |

Supplemental Table S2. Soil total carbon stock (Mg C ha^-1^) in Corn and switchgrass during 2023 after three and a half years of management.

| Depths |  | Soil total C (Mg C ha^-1^) | |
| --- | --- | --- | --- |
|  |  | Corn | Switchgrass |
| 0-10 |  | 27.40 a | 31.05 a |
| (10-20) |  | 25.32 a | 24.16 a |
| (20-30) |  | 22.08 a | 21.20 a |
| (30-60) |  | 44.04 a | 34.12 a |
| (60-90) |  | 23.95 a | 22.99 a |
| 0-90 |  | 142.79 A | 133.52 A |
|  |  |  |  |
| Crop |  | 0.23 |  |
| Depth |  | <0.001 |  |
| Crop x Depth |  | 0.092 |  |

Supplemental table S3: Analysis of variance (ANOVA) showing effects of main factors of the Crop and growing season (Year) on cumulative greenhouse gas emissions (CO_2_-C and N_2_O-N) at Urbana, IL during 2020-22022.

|  | CO_2_-C | N_2_O-N |
| --- | --- | --- |
| Crop | 0.002** | 0.014* |
| Year | 0.082 | 0.002** |
| Crop x Year | 0.006** | 0.242 |

*significant at p < 0.05; **significant at p < 0.01; ***significant at p < 0.001.

Supplemental table S4: Nitrate-N concentrations (means $\pm$ SE) in switchgrass and corn plots at 0-30 cm and 30- 90 cm depths in Urbana, IL, during the 2020-2022 growing seasons. Lowercase letters indicate the differences due to Year × Crop × Depth interactions. Uppercase letters indicate differences between crops when aggregated over Depths (rows) or Year (columns).

| Year |  | Corn | | |  | Switchgrass | | |
| --- | --- | --- | --- | --- | --- | --- | --- | --- |
|  |  |  | --------- NO_3_^-^ (mg L^-1^)---------------- | | | | | |
|  |  | 0–30 cm | 30–90 cm | Mean^1^ |  | 0 –30 cm | 30–90 cm | Mean |
| 2020 |  | 4.13 b | 4.23 b | 4.18 ABC |  | 3.56 b | 2.68 b | 3.12 BCD |
| 2021 |  | 9.88 a | 4.73 ab | 7.30 A |  | 1.09 b | 1.51 b | 1.3 CD |
| 2022 |  | 4.42 b | 5.40 ab | 4.91 AB |  | 1.70 b | 0.23 b | 0.97 D |
| Mean |  | 6.14 A | 4.78 A |  |  | 2.12B | 1.50 B |  |
| Source of  variation |  |  |  |  |  |  |  |  |
| Year |  |  |  |  |  |  | 0.196 |  |
| Crop |  |  |  |  |  |  | <0.001 |  |
| Depth |  |  |  |  |  |  | 0.105 |  |
| Year x Crop |  |  |  |  |  |  | 0.009 |  |
| Year x Depth |  |  |  |  |  |  | 0.288 |  |
| Crop x Depth |  |  |  |  |  |  | 0.555 |  |
| Year x Crop x Depth |  |  |  |  |  |  | 0.027 |  |
